# Europe’s New Identity: The Refugee Crisis and the Rise of Nationalism

**DOI:** 10.5964/ejop.v12i2.1191

**Published:** 2016-05-31

**Authors:** Claudia Postelnicescu

**Affiliations:** a University of Tübingen, Tübingen, Germany

Europe is nowadays at a crossroad, divided between the need to remain faithful to its core democratic values and freedoms, maintaining an area of freedom and justice and the need to protect its citizens against the new terrorism and the rise of nationalistic leaders and parties that require less Europe and more power back to the nation states. We are witnessing a return of the politics of fear; in Hobbes terms, convincing people that there is no other alternative and that politics has been exhausted; it remains the fear (Furedi, 2005). In the age of the war on terror, fear was a necessary argument to be brought on for justifying the US foreign policy in the Middle East (Robin, 2004). Nowadays, in the Middle East radical Islamism is justifying the war on the West through its faceless jihadist warriors spread across Europe, on the new hybrid terrorism that replaced wars on the ground. Hence, the enemy is becoming present and imminent, so the protectors arise justifying, once again, the language to legitimate new policies or public choices (Robin, 2004). The rise of extremism, radicalization and populism are facets of the same politics of fear. When the refugees arrived, the protectors against the identified enemy emerged.

In the following article the mechanisms used to prepare the society for the arrival of the populist Messiah will be demystified from a political science perspective and also analyzed from a legal point of view; the only bonding agent to a real European identity through various political disagreements is throughout law and the guiding common values. However the most critical factor in the success or failure of this type of leadership and in the seductive appeal of extremism is the emotional factor, the tactics employed to trigger the raw emotions present in the human nature particularly in crises. Beyond politics of fear we also need to look at and understand the politics and policies that took us here: the multicultural discourse with its policies of integration, assimilation and tolerance partially produced the adversary effect: a generation of young people who do not feel they belong, who do not wish to assimilate and who are not embracing either being tolerated or tolerate. They are the European jihadists, who took policy makers and politicians by surprise. To understand the psychology of these people will enable us to properly fight against the root causes of extremism, radicalization and terrorism; moreover, might lead us to understand what should be the adhesive of a genuine European identity.

Europe has always struggled with conflicting visions of its identity, of a unifying idea that will erase national particularities, a generous idea with such irreducible values (Hazard, 1989). We are witnessing now, after a long process of integration, a return to instinctive national sentiments. In the face of fear, people want to feel safe; hence a leader who can promise security and protection is gathering the popular support: we see it in the recent Austrian elections, where the far-right party of Norbert Hofer gained the upper hand and determined the resignation of the Chancellor amid huge debates over the refugee crisis. Nationalism is a crisis of identity (Smith, 2003), the response to the irregularities of modernity by taking pride in your own nation. The exhaustion of politics and modernity refuels the seduction of a puritan ideology, ready to sacrifice anything for the idea; the so-called Islamic State with its political religion in the name of certain purity opposed to modernity proved to have a terrible impact on the European youngsters joining by hundreds a foreign jihad. Puritanism and politics are not new in Islam and fundamentalist Wahhabism is the weapon against modernity and the West, a deadly one, using religion as the community aggregator against the identified enemy. However, marginal youth and a romantic version of nationalism appeared first in Europe in the creation of the nation states and later were exported as such in the European colonies (Kedourie, 1960/1993); from the clash between cultures and the shattered sense of self emerged generations of immigrants in Europe. Nonetheless we are observing now the same divided self and a confused identity in the third generation immigrants, young people becoming terrorist fighters, but at the same time a diverging vision about what is precisely to be European.

The dilemma, from a legal point of view, is profound, as the EU cannot function unless the EU law is implemented in the member states and creates a common ground for policies and legal measures in the face of crisis. The refugee crisis emphasized precisely the lack of common legal procedures. Although the Dublin agreement, revised a third time, was decided and agreed upon the member states in 2013, by the peak of the refugee crisis in the summer of 2015, the European Commission requested to a significant number of member states to make the necessary steps in order to translate the Dublin agreement into the national legislation. However, the current crises over a new Dublin, the forth, is just one issue where the divergent visions about the future of Europe come cross: the supranational, overly legalized and bureaucratic European Union that demands the national jurisdictions to harmonize with the EU law or a federal system where each state joins some of the agreements and refuses other.

## Europe’s Internal Conflict

The refugee crisis, with its millions of people fleeing war and conflicts, let aside the ones migrating to Europe for economic reasons, triggered the acceleration of an underlying conflict of visions among the European member states and even states outside the European Union, such as Iceland who dropped the plan of joining the European Union. There is suddenly a sense of urgency in dealing with all kinds of pressures inside the EU: some of those are old issues with new developing patterns, others are new: the inapplicability of the Dublin III agreement that underlined the minimal common rules on asylum-seekers and migrants failed implementation in several EU states in the summer of 2015, the financial crisis, the Greek debt crisis, the Crimeea/Ukraine crisis, the Brexit crisis, the terrorism crisis, with attacks or threats in Brussels, Paris or anywhere else in Europe. On the other side there is the rise of extremist parties in many European countries; the radicalization among European citizens who are becoming foreign terrorist fighters; the rise of hybrid terrorism and cyber terrorism; the end of Schengen area and the future of a more integrated EU.

The debate over these pre-existing aspects acquired more heat in the refugee crisis, when Angela Merkel went forward with an open gate approach, while other European countries refused to follow suit. The most prominent opponent of Germany’s vision is Hungary, through its prime-minister Viktor Orban, who refused any solidarity and proclaimed the demise of Schengen. The gap between Germany and other European States is widening also on a number of other issues, such as the Greek debt crisis and the Euro zone. The failure to narrow this gap might mean the disintegration of EU.

At the same time, the incapacity of the European leaders to prove solidarity by voluntarily taking in refugees generated a huge amount of pressure on the few countries that had no other choice. Greece and Italy are some of the main entry points in the EU and were put in the impossibility of facing the waves of refugees alone; in fact Greece did not manage to keep the Dublin requirements in place and large numbers of people went unregistered across Europe giving rise to criticism and anti-immigration phobias. From here to a full revival of nationalism and extreme right parties was only a step further.

What all these new leaders claim? They justify the return to the nation state and the national identity and the rejection of the union. What Europe claims? Divided visions, no common European identity and going from a crisis to another with no rational solution in sight. The situation is rapidly deteriorating with the success of nationalist leaders riding the wave of instability and fear of the future among huge migrants’ waves and imported terrorism in Europe via ISIL. In the background looms also the involvement of Russia’s president, Vladimir Putin, who is more than happy to see the European Union crumbling into pieces.

## Extremism and the Rise of the Populist Messiah

The European Union is divided. Recently a new phenomenon is contributing to the division: radicalization and terrorism. The disregarded youngsters are a source of unrest that obliged France to take measures for their reinsertion in society in an attempt to avoid and prevent future terrorist attacks organized by nationals; Paris with its 193 victims is looming over each European capital. De-radicalization programs are thought over and implemented also in UK, Denmark, Germany, Sweden, Netherlands, in order to change the narrative that drives so many young Europeans to the deadly ideology of political Islam and Jihad, offering their life to a foreign cause. Why are they seduced by such a simplistic worldview while often leading wealthy lifestyles? The suicide bombers are sometimes from wealthy families: young people looking to belong, searching for a certain identity, for something to fill a vacuum that their society, community or family cannot offer. This ideology and its success is plaguing Europe and is a deadly internal weapon that is challenging the very core of European values. There are also the marginal youngsters left out by the system who, despite all the opportunities of a Westernized lifestyle, they cannot fully engage in it, and then the resentment, anger and hate are ripe for being used by the preachers of jihad.

At the opposite side of the spectrum are those who radicalize their discourse against immigration, refugees or anyone who is foreign. The psychology involved in this is simple: you turn outwards your own frustrations with the way things are as an emotional coping mechanism. In this air of tension both extremists and populist false Messiah thrive: an apparent revolutionary that will change the way things are. People are asking for solutions and a radical change and they will promise unscrupulously anything. The discourse is simple: appealing to national values, national identity, national borders, and national interests. Viktor Orban in Hungary, who built a wall against refugees and triggered a collapse on the Balkans’ borders, Miro Cerar in Slovenia who said that his country will only accept Christian refugees and that Europe is going to drown, Norbert Hofer, the far-right leader in Austria that promised he will protect Austrian borders against the refugees. The all have a palpable enemy: the refugee. What is then the difference between the hostility towards the refugee and Hitler’s anti-Jewish hate? Hate is a unifying factor in all mass movements (Hoffer, 1951), is the common denominator capable of putting together the most different people.

## The Fear Factor and the Profile of Radicals

The evocation of fear and pointing to a real or imagined enemy that must be hated and fought against is common among both radical Islamists and populist demagogues in the United States and Europe. The ground being set for a change in politics, the hate speech is the most powerful vector: from the disillusioned second or third generation of young immigrants in the West to the youngsters of the Middle East, the hate against the identified enemy fuels a dangerous war, a non-conventional war, with insidious hybrid means of proliferation: cultural, cyber, social media, and lone wolf terrorism.

While Donald Trump rages against the Mexican immigrants alike with Muslims, in Europe the enemy has rapidly become the refugee, while in the Middle East the enemy is the cultural West with its decadent lifestyle and its Christian religion. We may call this an ideology of fear, as it contains forms of other ideologies: Islamism, fascism, anti-Semitism, all ideologies based on exercising violence as a political tool. The appeal to emotions and the facility of creating a persona through media renders democracy extremely vulnerable to populism and nationalism. It is what Plato (380 B.C./2010) identified centuries ago that despots arise from later stages of democracy when equality abolishes all limitations and liberties are at their peak. 

Strategic opportunism in a democracy poses fundamental challenges and sets the ground for tyranny (Ostrom, 1997). The tough abrasive leader that offers solutions to all problems of society and exploits the potential of fear to its maximum should not be difficult to be identified. They appeal to the masses and speak their language using emotions and empowered them with their persona. We have seen recently how Trump’s supporters took violence to make their point against the dissenters. Trump himself has no shame in spreading violent messages against Muslims, women and others, endorsing torture and promising to solve jihadist terror once and for all; anything goes once you got the popular endorsement with emotions, not rationality. In Europe, popular militias of extreme-right are hunting down immigrants and Muslims, justifying their actions through the idea of security and protection for fellow citizens and Christians. Media reports about migrants’ assaults or attacks generate, in a mirror effects, the violent response for the other extremists, arising conflict and changing the public perceptions in the lack of governmental solutions.

## Possible Outcomes in the European Union

In the recent European elections the far right has risen with populist slogans and an anti-immigration and anti-European discourse. In Austria the far right party, FPŐ won the first round of presidential elections, which places Norbert Hofer, their leader in top position of becoming the president. In Poland, the extreme conservative party won the elections and drove large mass protests recently; the discourse is the same, anti-immigration and the pride of national identity. In France, Marine Le Pen is openly pro Russia and expressing a strong anti-European sentiment. The idea of being proud of your nation against the abstract bureaucracy of Brussels is exploiting also against whoever is not a citizen. Not only that some Europeans are against others, but the openness and humanity that stood as core European values are strained now in the new political climate dominated by fear and lack of security. 

The terrorist threats and the attacks in the heart of Europe, in Paris and Brussels, augment the seducing power of a Messianic leader or party. All the populist new leaders are talking about protecting nationals against foreigners, refugees and other Europeans alike, and about the abuses in the European Union. Suddenly the Union is not appealing and is not a unifying force, which renders Germany as the main motor of further integration, with France on its side. However the fracture between Eastern and Western Europe revives. Unfortunately there is an ongoing talk about two-speeds Europe or, even worse, a la carte Europe, where any member states gives and takes as much as it wants. As a consequence of this approach, common legal standards among European states will be extremely difficult to set in place and that is the case with the Dublin Agreement.

Nationalist, anti-liberal and anti-European parties are gaining ground all over in the European Union member states. The impasse with the Schengen agreement is pointing into a rather stressful European future: the German change of tone towards the Central and Eastern European states that refused to show real solidarity in the refugee crisis reflects a deeper divergence that will echo on the common European security agenda and migration policy. The difficulty of reaching an agreed plan with solutions for the current migration crisis, despite a series of several meetings in the European Council, reveals profound discrepancies in internalizing core European values and a different degree of attachment to the European project and idea.

In Europe, the debate about a democratic deficit within European Union is pretty old; the Commission developed a series of programs and open consultations with citizens in order to cover the gap between the political elite and the electors. Despite these efforts, there is a sustained trend in Europe against the classical political parties; people are organizing their own parties, representing a certain community (local or a community of interests, such as the Pirates Party, who managed to get their representatives in the European Parliament). Also many outsiders, either from civil society, entrepreneurship or business join politics as independents and getting the popular vote. There seems to be a strong rejection against full-time politicians, particularly those active for a long time. Citizens tend to favor more and more a new generation of leaders, people with a clean background, uncompromised in entanglements of corruption accusations or political failures. Hence, the rise of a populist, Messianic leader that speaks with the precise scope of seducing the electors with false promises of solving all their problems is already notable. In the US preliminary elections of the candidate that will remain in the presidential competition, to anyone’s surprise Donald Trump outweighed his Conservative opponents through blatant populism, racism, fear-mongering and irreverence, using the politics of fear to gain electoral profits.

## Concluding Remarks

Europe needs to find its identity and unique voice, not many independent and divisive voices if wants to survive in the new European and global environment: it needs to stand united against Putin’s Russia and his gambling in Ukraine, Middle East and Syria. Europe’s voice should include all citizens and the new comers alike, as being a refugee is respected through international and European standards. Europe is already intercultural and it will remain so, but the fear and hate need to be kept out of the public discourse. However, until now, Europe did not manage to offer a sense of common identity beyond the common values and an idea of Europe, which maintains its “mixed identity” (Said, 1995).

If Europe decides to move on in two speeds or even worse to compromise on a Brexit, the whole castle will crumble; the long process of integration will disintegrate. Europe, in the context of many geo-political and hybrid threats cannot afford that and each member state will remain weak on its own. However, to remain united, compromises must be agreed and implemented, and this had become increasingly difficult. The major challenge for Europe is to find the proper balance between all these conflicting needs: security, freedom and unity. The crises are also an opportunity and the much need deeper integration should counter-balance the existing choices.

The war is already inside: it is first and foremost a war of visions and a war for dominating power in the international arena for the coming years; former alliances are shifting and new players arrived. Russia has gained new force as a strategic global power in the Syrian conflict and by getting away with the infringement of international law when invaded Ukraine. ISIL is the new tool of many states for maintaining war and chaos in the Middle East and also in keeping a refugee crisis for Europe. The war is on and is involving not only political disagreements but cultural crises, and resuscitates the old hate against Occident and modernity with its ideological fiction. At the same time, the war is inside of us, Europeans, for now knowing truly who we are and who we are ought to be.

Future interesting research might look at this very tricky psychological contraction that is pulling us, Europeans, to negate our heritage, our Christianity, our lifestyle, our freedoms in order to accommodate and make home for those who are not born Europeans, yet we fail: we fail us and we also fail them, the others; there seems to be no escape. Perhaps, in our quest to celebrate diversity we didn’t first answer a few crucial questions: who we are and what are we, Europeans, stand for and how? What makes Europe strong and what makes Europe weak? Why our young Europeans feel depressed and abandoned and are they so easily seduced by an ideology of death and destruction? How can Europe welcome others when among us, Europeans, we are so divided? The solutions to our crises are not only political or legal, but within us, in the way we internalize our identity, particularly in the meeting with the other, enemy or not and that is certainly an old dilemma.
